# CA19.9 Serum Level Predicts Lymph-Nodes Status in Resectable Pancreatic Ductal Adenocarcinoma: A Retrospective Single-Center Analysis

**DOI:** 10.3389/fonc.2021.690580

**Published:** 2021-05-27

**Authors:** Alessandro Coppola, Vincenzo La Vaccara, Michele Fiore, Tommaso Farolfi, Sara Ramella, Silvia Angeletti, Roberto Coppola, Damiano Caputo

**Affiliations:** ^1^ Department of Surgery, Campus Bio-Medico University, Rome, Italy; ^2^ Radiation Oncology, Campus Bio-Medico University, Rome, Italy; ^3^ Unit of Clinical Laboratory Science, Campus Bio-Medico University, Rome, Italy

**Keywords:** pancreatic cancer, carbohydrate antigen 19.9, pancreatic surgery, lymph node staging, albuminemia, margin status

## Abstract

**Background:**

The choice between upfront surgery or neoadjuvant treatments (NAT) for resectable pancreatic ductal adenocarcinoma (R-PDAC) is controversial. R-PDAC with potential nodal involvement could benefit from NT. Ca (Carbohydrate antigen) 19.9 and serum albumin levels, alone or in combination, have proven their efficacy in assessing PDAC prognosis. The objective of this study was to evaluate the role of Ca 19.9 serum levels in predicting nodal status in R-PDAC.

**Methods:**

Preoperative Ca 19.9, as well as serum albumin levels, of 165 patients selected for upfront surgery have been retrospectively collected and correlated to pathological nodal status (N), resection margins status (R) and vascular resections (VR). We further performed ROC curve analysis to identify optimal Ca 19.9 cut-off for pN+, R+ and vascular resection prediction.

**Results:**

Increased Ca 19.9 levels in 114 PDAC patients were significantly associated with pN+ (p <0.001). This ability, confirmed in all the series by ROC curve analysis (Ca 19.9 ≥32 U/ml), was lost in the presence of hypoalbuminemia. Furthermore, Ca 19.9 at the cut off >418 U/ml was significantly associated with R+ (87% specificity, 36% sensitivity, p 0.014). Ca 19.9, at the cut-off >78 U/ml, indicated a significant trend to predict the need for VR (sensitivity 67%, specificity 53%; p = 0.059).

**Conclusions:**

In R-PDAC with normal serum albumin levels, Ca 19.9 predicts pN+ and R+, thus suggesting a crucial role in deciding on NAT.

## Introduction

Pancreatic ductal adenocarcinoma (PDAC) is a leading cause of cancer-related deaths. Moreover, according to the projections, it has been estimated that pancreatic cancer incidence is increasing (1).

Surgical resection represents the optimal treatment for patients affected by PDAC (2, 3).

Unfortunately, due to the advanced stage of the disease at first diagnosis, mostly related to the presence of distant metastases or local vascular invasion, only a minority of PDAC patients are eligible for upfront surgery (4).

For this reason, several different programs for pancreatic ductal adenocarcinoma early detection have been evaluated to date, yet it remains a hot-topic for research to improve outcomes. In this field, recent advances are showing promising results in the development of cheap and user-friendly tools, also based on the use of emerging technologies such as nanotechnology (5, 6).

Regrettably, despite being very promising (7), these technologies are still far away from being validated for routine daily clinical use.

Moreover, even for patients selected for radical resection, the prognosis remains poor due to nodal involvement and the higher rates of local or distant recurrences. In this regard, actual 5-year low survival rates of 17% have been reported, with the grade of lymph node involvement proving to be among the main predictors of survival in resected PDACs (8)

For these reasons, chemotherapy and chemo-radiation therapy are often proposed in a neoadjuvant setting, aiming to increase the rate of resectability and achieve better oncological results (9, 10).

Since pancreatic surgery is often burdened by severe morbidity and not-negligible mortality, even in high-volume centers, resected patients often experience a delay in starting adjuvant treatments with consequent impairment of long-term outcomes. Therefore, some authors proposed the use of minimal-invasive approaches with the hope of decreasing postoperative morbidity and reducing the time between surgery and adjuvant treatments (11).

Currently, NAT is offered mainly in borderline resectable and locally advanced pancreatic cancers as it seems to offer a higher rate of negative pathological nodal status, as well as negative margins status (10, 12–14).

In addition to the above-mentioned oncological advantages, it has also been reported that during NAT the performance status of patients with jaundice and/or with relevant weight loss can be improved. Furthermore, Raufi claimed better local control for NAT due to the absence of vascular flow change derived from surgical resection (15).

Due to the lack of reliable tools to predict nodal involvement, some authors suggest the use of NAT in all patients with pancreatic cancer (16). Unfortunately, inversely, NAT toxicity can preclude surgery for patients who may potentially have no lymph node involvement.

As reported by Gaskill, NAT can result in the patient losing “the window for surgical cure” (17).

Both computed tomography (CT scan) and magnetic resonance imaging (MRI) can classify pancreatic cancer in four main categories: resectable (R), borderline resectable (BR), locally advanced (LA), and metastatic (UR) (18). This classification is based on local vascular invasion, as well as on the presence of parenchymal and peritoneal metastases, and this radiological staging identifies patients either for upfront surgery or for oncological treatments (19, 20).

In this scenario, the availability of tools that can predict nodal involvement before any treatment would be desirable.

Carbohydrate antigen 19.9 (Ca 19.9) is the only marker approved for clinical use by the Food and Drugs Administration (FDA) for PDAC, especially in the postoperative follow-up period. Unfortunately, the low specificity (82%; range 68–91%) and sensitivity 79% (70–90%) prevent the use of Ca 19.9 in the first staging of patients (21).

However, Ca19.9 assessment has been reported to improve patient selection for neoadjuvant therapies or upfront surgery, followed by adjuvant chemotherapy (22).

Furthermore, Ca 19.9 demonstrated its efficacy in combination with PET-CT scan in predicting progression and overall survival in locally advanced PDAC submitted to chemotherapy and radiotherapy (23).

Nonetheless, in a cohort of 160 PDACs, Wang also demonstrated that the association between the findings of 18F−FDG PET/CT (SUV *≥7.05*) and Ca 19.9 (levels ≥*240.55*) significantly predicted nodal micrometastases (24).

More recently, Hua proposed a nomogram based on the use of Ca 19.9 and other markers (Ca 125, Ca 50 and Ca 242), able to predict nodal positivity in PDAC (25).

To the best of our knowledge, the validity of Ca 19.9 in predicting nodal involvement in patients affected by PDAC has not been investigated so far.

Nevertheless, nutritional and inflammatory biomarkers (e.g., albumin, C-reactive protein CRP, neutrophils, etc.) (26, 27) and standard laboratory tests (e.g., hemoglobin, bilirubin, etc.) (28) have been reported as useful, alone or in combination, and with Ca 19.9, in assessing the prognosis of PDAC patients.

Chen and colleagues (29) demonstrated that neutrophil to lymphocyte ratio (NLR), CA19.9 levels, and the presence of circulating regulatory T cells are significantly associated with overall survival in patients with resectable pancreatic cancers. These findings support the theory that systemic inflammation and immune system disorders are strictly associated with the development and spread of different neoplasms, including pancreatic cancer.

Shuai-Shuai Xu (30), investigating the role of standard laboratory tests, such as hemoglobin, albumin, and blood cell count, demonstrated that the combination of hemoglobin, albumin, lymphocyte and platelet (i.e., HALP), was helpful in assessing the oncological outcome of patients who underwent radical resection for PDAC.

Moreover, considering that serum albumin, levels reflect the nutritional status of PDAC patients, and in line with evidence that an advanced disease is more likely to be associated with cachexia, special attention has been given recently to the link between Ca 19.9 and serum albumin levels in assessing prognosis of PDAC. In Zhang’s experience, lower levels of serum albumin and higher levels of Ca 19.9 have been reported to be associated with a more severe prognosis in stage III PDAC (31).

The main aim of this study was to evaluate the role of Ca 19.9 serum levels in predicting nodal status in R-PDAC.

The primary scope of this observational retrospective cohort study was to analyze the role of Ca 19.9 in predicting the nodal involvement in patients affected by PDAC. Moreover, the effect of serum albumin level (SAL) on Ca 19.9 efficacy in this regard was also evaluated. The secondary objective of the study was to evaluate the link between Ca 19.9 serum levels and the resection margin status, and the need for a vascular resection.

## Materials and Methods

Data collected from a prospective database of patients who underwent resective pancreatic surgery at the University Hospital Campus Bio-Medico of Rome between June 2000 and December 2019 has been retrospectively analyzed. The present study was approved by the Local Ethical Committee of the University Campus Bio-Medico of Rome (protocol number 104.20 OSS ComEt CBM) and performed in accordance with the Declaration of Helsinki and in respect of the principles of good clinical practice.

Inclusion criteria were age ≥18 years old, diagnosis of pancreatic adenocarcinoma, tumor radiologically staged as resectable by the multidisciplinary pancreatic cancer board of our institution, with available preoperative serum albumin and Ca19.9 levels. Exclusion criteria were: patients aged <18 years, unavailable preoperative SAL and Ca19.9 levels, history of previous NAT, or any other oncological treatment.

“Margin status after surgery was defined as negative (R0) in case of free margin ≥1 mm, or positive (R1) in case of free margin <1 mm, and R2 in case of macroscopic tumor invasion (32).

General demographic characteristics including age, sex, obesity (defined as Body Mass Index; BMI ≥30 kg/m^2^), comorbidities, American Society of Anesthesiologists score (ASA), and cancer characteristics as tumor location, dimension (T stage), presence of nodal involvement (N+), resection margins positivity (R+), and need of vascular resections (VR), were evaluated. All the pathological reports were re-staged according to the TNM classifications proposed by 7th edition classification of The American Joint Committee on Cancer and the International Union for Cancer Control (33).

According to our laboratory values, hypoalbuminemia was considered for SAL <3.2 gr/dl, while Ca19.9 was considered elevated for ≥37 U/ml.

### Statistical Analysis

In the first phase, an analysis was performed considering local laboratory values for Ca 19.9 and serum albumin level. For continuous and categorical variables (age, sex, obesity, diabetes, ASA, Tumor location, tumor stage, positive lymph-nodes, positive margins and vascular resection) the χ2 test for proportions was used, p values <0.05 were considered significant.

Multiple logistic regression was used for multivariate analysis performed a using the following dependent variables: nodal status, margin positivity and the need for vascular resection. As independent variables, Ca 19.9, albumin levels, each single pT stage and tumor grading were considered. Nodal status, margin positivity, and need for VR were independent variables when not used as dependent ones. Data was analyzed using Med-Calc 11.6.1.0 statistical package (MedCalc Software, Mariakerke, Belgium). All probabilities were two-tailed, and p values ≤0.05 were regarded as significant.

In the second phase of the analysis, Receiver Operating Characteristic (ROC) analysis was performed, and Area under the Curve (AUC) was calculated to define the cut-off point for the Ca 19.9 marker and its accuracy in node positivity prediction, margin involvement, and vascular resection in patients with normal and low serum albumin levels. The odd ratio has been computed to investigate if a Ca 19.9 level higher than the established cut-off could identify patients at significant risk for N positivity and complications (34).

## Results

**Figure 1:**
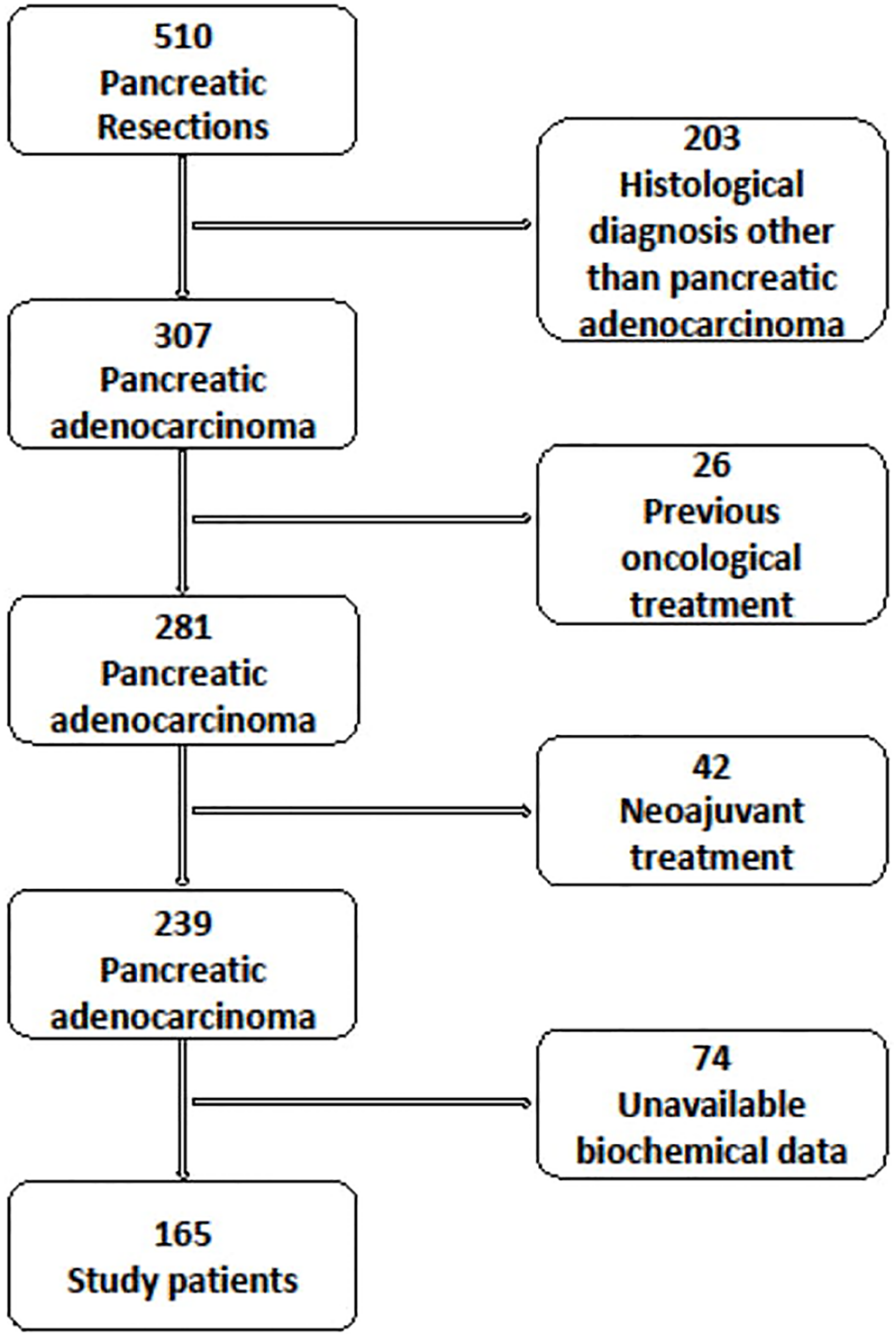
Flow-chart showing selection process.

Of the 510 PDAC patients resected in the period, 165 fulfilled the study inclusion criteria (**Figure 1**).

The demographic and tumor characteristics of all the series have been reported in **Table 1**.

**Table 1:** Series demographics and tumor characteristics.

	Total Patients 165 N (%)
**Age (median)**	70 (range 42–85)
**Sex**	
** Male**	95 (57.5%)
** Female**	70 (42.5%)
**Obesity BMI ≥30 kg/m^2^**	9 (5.4%)
**Diabetes**	20 (12.1%)
**ASA classification**	
** 1–2**	76 (46%)
** 3–4**	89 (54%)
**Tumor Location**	
** Head**	131 (79.4%)
** Body-Tail**	34 (20.6%)
**T Stage**	
** T1**	10 (6%)
** T2**	35 (21.2%)
** T3**	120 (72.8%)
**Positive lymph-nodes (N+)**	122 (73.9%)
**Stage**	
** I**	22 (13.3%)
** I A**	8 (36.4%)
** I B**	14 (63.6%)
** II**	143 (86.7%)
** II A**	21 (14.7%)
** II B**	122 (85.3%)
**Positive Margin (R+)**	65 (39.3%)
**Tumor Grading**	
** G1**	19 (11.5%)
** G2**	105 (63.6%)
** G3**	36 (21.9%)
** Gx**	5 (3%)
**Median number of harvested lymph-nodes**	31 (range 3–84)
**Median number of positive nodes**	3 (range 1–57)
**Median LNR**	0.09 (range 0.01–0.84)
**Ca 19.9 median levels (range) U/l**	109.8 (0.76–15,400)

ASA, American Society of Anesthesiologists; BMI, Body Mass Index; LNR, lymph-node ratio (lymph-node positive/total of lymph-nodes).

According to the size of the tumor and final pathological stage, T3 pancreatic cancers were more represented. In detail, pT1 was staged in 10 cases (6%), pT2 in 35 cases (21.2%) and pT3 in 120 cases (72.8%).

The lymph nodal involvement was detected in 122 patients, which represented 73.9% of the whole series.

Resection margin positivity was identified in 65 patients (39.3%).

Ca19.9 levels were under the normal laboratory values (i.e., <37 U/ml) in 51 cases (31.3%). This group of patients has been identified as the CN Group. Elevated (i.e., ≥37 U/ml) Ca 19.9 levels have been registered in 114 patients (68.7%). These patients have been categorized as the CH Group. The two groups were homogenous with regard to age and the prevalence of obesity and diabetes. No significant differences have been found in terms of ASA score and tumor location.

As shown in **Table 2**, a significantly higher rate of female (49.12% vs 27.5%, p = 0.009) and T3 stage (78.1% vs 60.9%, p = 0.02) were detected in the CH Group (**Table 2**), while T1 PDAC were significantly higher in the CN Group (11.7% vs 3.5%, p = 0.04).

**Table 2:** Demographics and tumor characteristics according to normal or elevated Ca 19.9 levels.

	Ca 19.9 <37 U/ml 51 Patients n (%)	Ca 19.9 ≥37 U/ml 114 Patients n (%)	p value
**Age median**	71 (range 44–84)	69.5 (range 42–85)	0.55 *ns*
**Sex**			
** Male**	37 (72.5%)	58 (50.9%)	**0.009**
** Female**	14 (27.5%	56 (49.1%)	
**Obesity BMI ≥30 kg/m^2^**	1 (1.9%)	8 (7%)	0.18 *ns*
**Diabetes**	3 (5.8%)	17 (14.9%)	0.10 *ns*
**ASA classification**			
** 1–2**	23 (45%)	53 (46.4%)	0.86 *ns*
** 3–4**	28 (55%)	61 (53.6%)	
**Tumor Location**			
** Head**	43 (84.3%)	88 (77.2%)	0.26 *ns*
** Body-Tail**	8 (15.7%)	26 (22.8%)	
**T Stage**			
** T1**	6 (11.7%)	4 (3.5%)	**0.04**
** T2**	14 (27.4%)	21 (18.4%)	0.18 *ns*
** T3**	31 (60.9%)	89 (78.1%)	**0.02**
**Stage**			
** I**	15 (29.4%)	7 (6.1%)	<0.0001
** I A**	6 (40%)	2 (28.6%)	0.60 *ns*
** I B**	9 (60%)	5 (71.4%)	<0.0001
** II**	36 (70.6%)	107 (93.9%)	0.13 *ns*
** II A**	8 (22.2%)	13 (12.1%)	
** II B**	28 (77.8%)	94 (87.9%)	
**Positive lymph-nodes (N+)**	28 (54.9%)	94 (82.4%)	**0.0002**
**Positive Margin (R+)**	16 (31.3%)	49 (42.9%)	0.15 *ns*
**Vascular Resections**	9 (17.6%)	33 (28.9%)	0.12 *ns*

Ca 19.9, Carbohydrate antigen 19.9; ASA, American Society of Anesthesiologists; BMI, Body Mass Index; NS, not significant. Underlined Bold values means statistical significant.

### First Phase

In all the series, the CH Group showed a statistically significant higher rate of N+ (82.4% vs. 54.9%, p <0.001), while no differences were observed in terms of R+ and VR (**Table 2**).

Patients in the CH Group showed a 3-fold higher probability of nodal involvement (OR 3.588) with a positive predictive value (PPV) of 79.85 and a negative predictive value (NPV) of 49% (p = 0.0008).

According to the preoperative albumin levels, SAL <3.2 gr/dl was detected in 63 patients. In this group, defined as L-SAL Group, patients were older (72 years vs 67 years, p = 0.027) and ASA score grades 3 and 4 were significantly more represented (66.6 and 46% respectively, p = 0.009). No statistically significant differences were detected in the two groups, in line with the prevalence of obesity and diabetes. The N-SAL Group showed a significantly higher tumor rate located in the body or the tail of the pancreatic gland (12.6% vs. 25.5%, p = 0.04). According to the pathological stage, no significant differences were found in terms of T stage. SAL did not show any significant association with the N, R+ and the need for VR (**Table 3**).

**Table 3:** Demographics and tumor characteristics according to Serum Albumin Level.

	Serum Albumin Level <3.2 g/dl 63 Patients n (%)	Serum Albumin Level ≥3.2 g/dl 102 Patients n (%)	p value^*^
**Age**	72 (range 44–85)	67 (range 42–84)	**0.027**
**Sex**			
** Male**	41 (65%)	54 (52.9%)	0.12 *ns*
** Female**	22 (35%)	48 (47.1%)	
**Obesity BMI ≥30 kg/m^2^**	6 (9.5%)	3 (2.9%)	0.07 *ns*
**Diabetes**	9 (14.2%)	11 (10.7%)	0.50 *ns*
**ASA**			
** 1–2**	21 (33.3%)	55 (53.9%)	**0.009**
** 3–4**	42 (66.7%)	47 (46.1%)	
**Tumor Location**			
** Head**	55 (87.3%)	76 (74.5%)	**0.04**
** Body-Tail**	8 (12.7%)	26 (25.5%)	
**T Stage**			
** T1**	4 (6.3%)	6 (5.9%)	0.90 *ns*
** T2**	12 (19.1%)	23 (22.5%)	0.59 *ns*
** T3**	47 (74.6%)	73 (71.6%)	0.67 *ns*
**Stage**			
** I**	7 (11.1%)	15 (14.7%)	0.50 *ns*
** I A**	3 (42.9%)	5 (33.3%)	0.66 *ns*
** I B**	4 (57.1%)	10 (66.7%)	0.50 *ns*
** II**	56 (88.9%)	87 (85.3%)	0.91 *ns*
** II A**	8 (14.3%)	13 (14.9%)	
** II B**	48 (85.7%)	74 (85.1%)	
**Positive lymph-nodes (N+)**	48 (76.1%)	74 (72.5%)	0.60 *ns*
**Positive Margin (R+)**	28 (44.4%)	37 (36.2%)	0.29 *ns*
**Vascular Resections**	12 (19%)	30 (29.4%)	0.19 *ns*

χ^2^ test for proportions. Underlined Bold values means statistical significant. NS, not significant.

Notably, Ca 19.9 serum levels were significantly associated with N+ (p <0.001) when serum albumin was normal.

In this subgroup of patients, higher Ca 19.9 levels were also significantly associated with higher rates of VR (p = 0.03). Notably, these findings were not confirmed in the case of hypoalbuminemia (**Table 4**).

**Table 4:** Nodal status, margin status and need for VR according to Serum Albumin and Ca 19.9 levels.

		Vascular Resection (VR) n (%)	Positive Lymph nodes (N+) n (%)	Margin Status(R+) n (%)
**Albumin Value ≥ 3.2 gr/dl**	102 Patients			
**CA 19.9 < 37 U/ml**	36 (35.3%)	6 (16.6%)	19 (52.7%)	10 (27.7%)
**CA 19.9 ≥ 37 U/ml**	66 (64.7%)	24 (36.3%)	55 (83.3%)	27 (40.9%)
		*p= 0.03	*p< 0.001	*p= 0.18 ns
**Albumin Value < 3.2 gr/dl**	63 Patients			
**CA 19.9 < 37 U/ml**	15 (23.8%)	3 (20%)	9 (60.0%)	6 (40.0%)
**CA 19.9 ≥ 37 U/ml**	48 (76.2%)	9 (18.7%)	39 (81.2%)	22 (45.8%)
		*p= 0.91 ns	*p= 0.91 ns	*p= 0.69 ns

Ca 19.9, Carbohydrate antigen 19.9; *^2^ test for proportions; NS, not significant. Bold values means statistical significant.

At the multivariate analysis, Ca 19.9 >37 and pT3 result was associated with significantly higher odds of lymph-nodes positivity. These results are shown in **Table 5**.

**Table 5:** Multivariate analysis and Odds Ratio demonstrating the ability of Ca 19.9 and pT3 in predicting the nodal positivity.

	Odds Ratio	Confidence Interval	P value
**CA 19.9 ≥37 U/ml**	3.0187	(1.2999–7.0102)	0.0102
**pT3**	7.1275	(2.9207–17.3934)	<0.0001

Other independent variables included in the model that did not reach statistical significance: Albumin <3.2 g/dl, tumor grading (G1, G2, G3), margin status (positive and negative), pT1, pT2, need for VR.

Ca 19.9, Carbohydrate antigen 19.9.

At the multivariate analysis, Ca 19.9 was not found to be an independent predictor of margin positivity and need for VR.

### Second Phase

**Figure 2:**
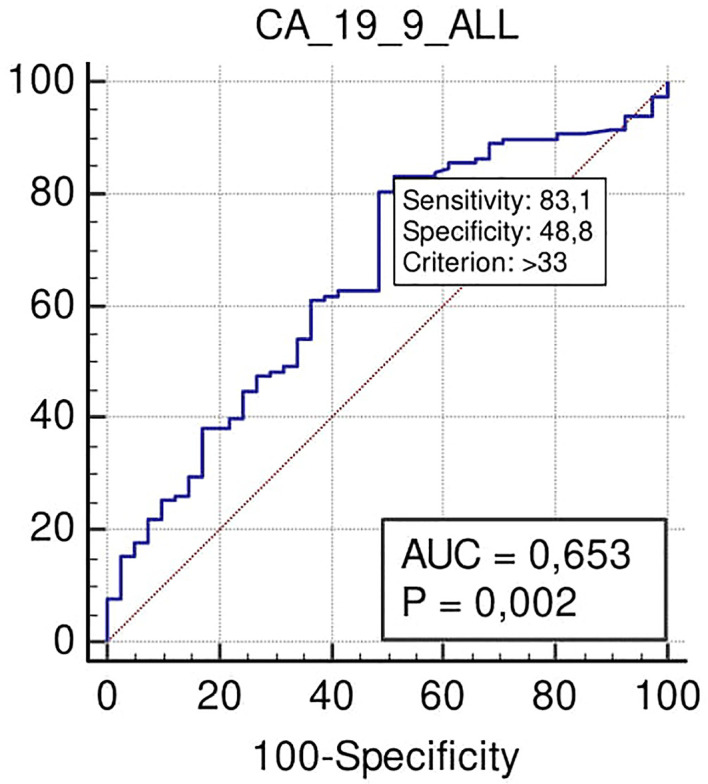
ROC curve analysis to define the optimal cut-off point for Ca19.9 (Carbohydrate antigen 19.9); accuracy in nodal positivity prediction in all the series.

ROC curve analysis performed in all the series showed how Ca 19.9, at the cut-off >33 U/ml, was associated with N+ with a sensitivity of 83%, a specificity of 43% (p = 0.002), a positive predictive value (PPV) of 80%, and a negative predictive value (NPV) of 50% (**Figure 2**).

**Figure 3:**
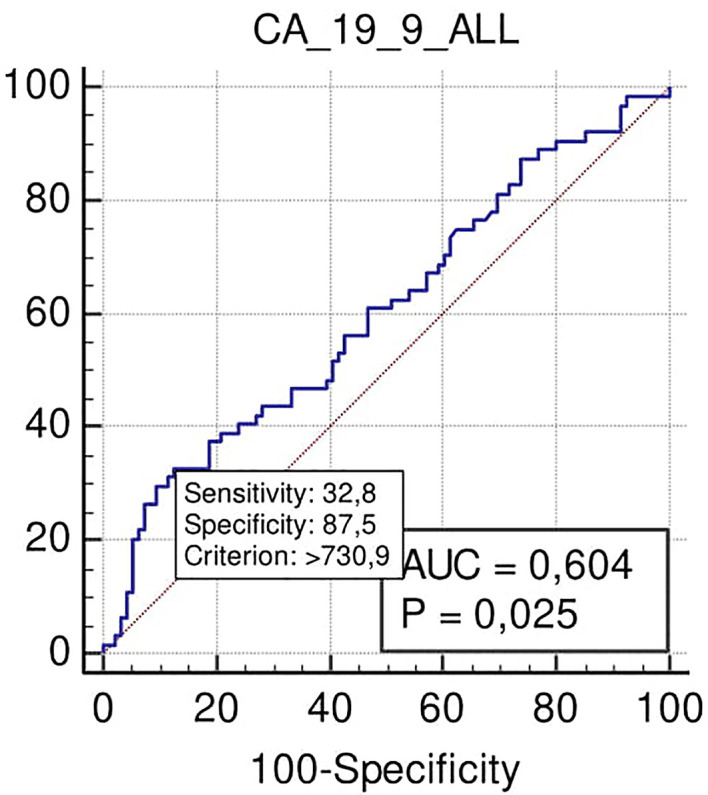
ROC curve analysis to define the optimal cut-off point for Ca19.9 accuracy in margins positivity prediction in all the series.

No significant association was found with the need for VR. On the contrary, margin positivity after surgical resection was observed for Ca 19.9 at the cut-off >730 U/ml (specificity 85%; p = 0.025), despite low PPV and NPV (63 and 66% respectively) (**Figure 3**).

**Figure 4:**
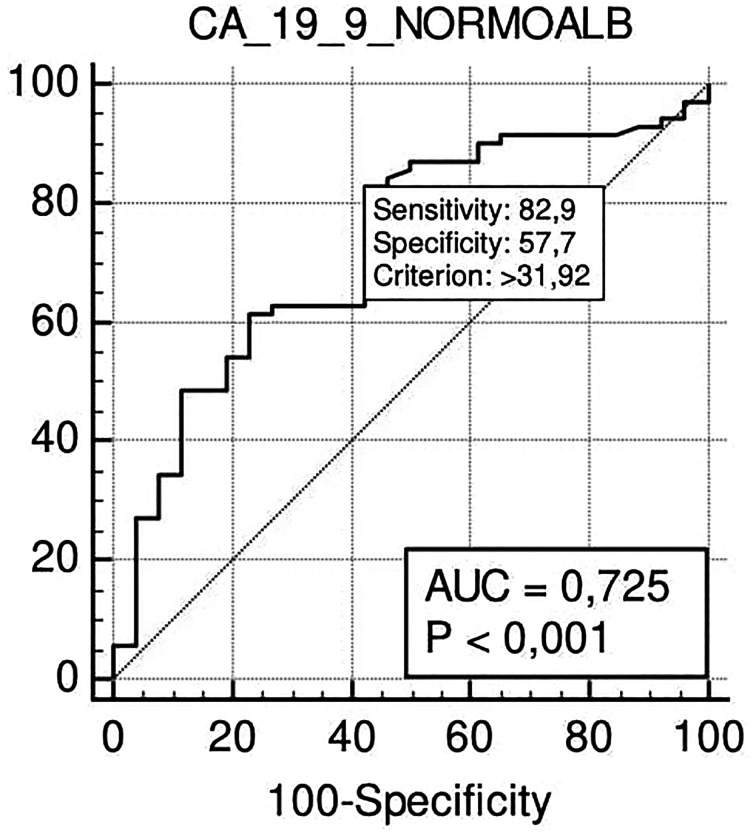
ROC curve analysis to define the optimal cut-off point for Ca19.9 accuracy in nodal positivity prediction in the N-SAL group.

In the N-SAL group, the ROC curve analysis confirmed that Ca 19.9 at the cut-off level of 32 U/ml was able to predict lymph nodal positivity with a sensitivity of 83% and specificity of 57% (p <0.001) (**Figure 4**).

**Figure 5:**
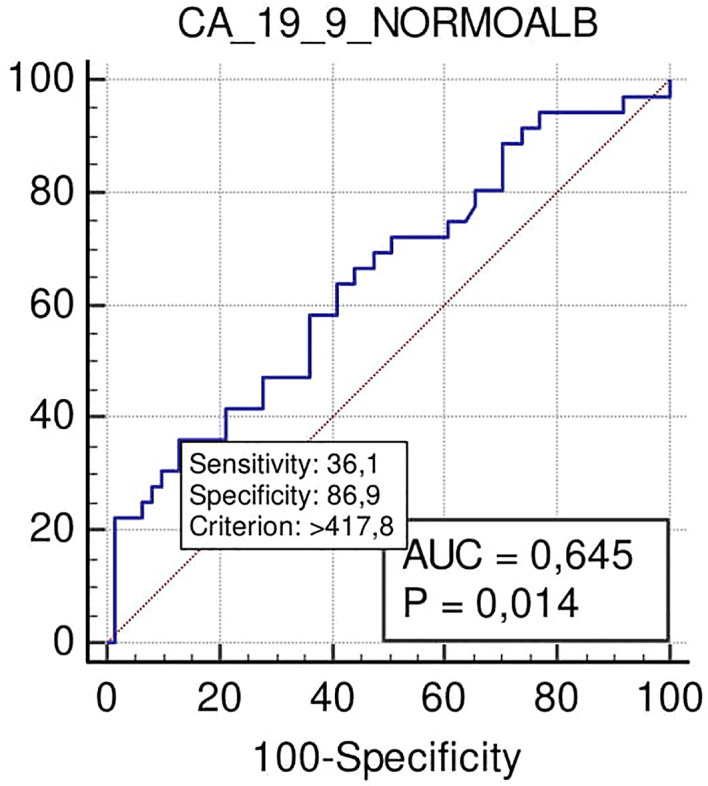
ROC curve analysis to define the optimal cut-off point for Ca19.9 accuracy in margin status prediction the N-SAL group.

At the cut-off level >418 U/ml, Ca 19.9 also predicted positive margins after surgical resection with high specificity (87%) and low sensitivity (36%) (p = 0.014), PPV 58%, and NPV 68% (**Figure 5**).

**Figure 6:**
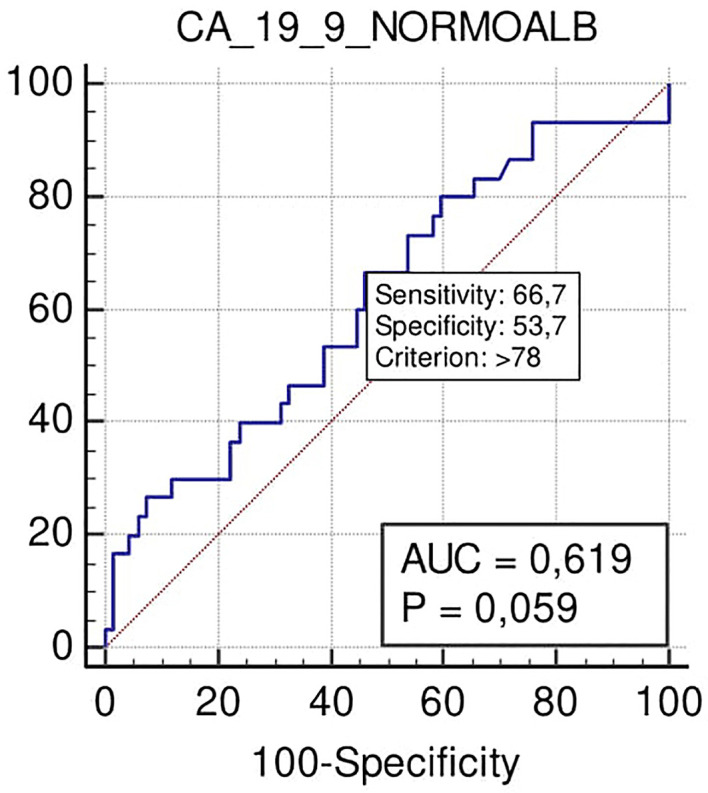
ROC curve of the role to predict VR of Ca 19.9 in patients with PADC in the normal serum albumin level group.

Notably, according to ROC curve analysis, Ca 19.9 at the cut-off >78 U/ml showed a significant trend for predicting the need for VR (sensitivity 67%, specificity 53%; p = 0.059) (**Figure 6**).

In the L-SAL group, ROC curve analysis notably showed that Ca 19.9 failed to predict N+, R+ and the need for VR.

## Discussion

Radical resection retains a fundamental role in the treatment of PADC, still representing the optimal standard of care.

PADCs are commonly defined as Resectable (R), Borderline Resectable (BR), Locally Advanced (LA), or Un-resectable (UR), based on radiological findings performed for tumor staging (19).

This classification is mainly attributed on the basis of the presence of local vascular invasion or distant metastases detected during the radiological clinical staging.

According to Kim (35), almost 20% of “presumed” R-PDACs have unexpected advanced disease status or occulted metastases.

Considering the promising results obtained after NT in patients with advanced disease, some authors propose chemotherapy before surgery even in patients who may be candidates for first-line resection (3, 14).

Consequently, upfront surgery for R-PDAC has now become controversial, especially in cases with lymph nodal involvement that could mostly benefit from NT.

Moreover, the Heidelberg group recently reported how lymph nodal involvement is the main factor impacting the survival of patients affected by pancreatic cancer (7). In this study, performed on a series of 937 patients, the number of involved lymph nodes was identified as the strongest prognostic factor for long-term oncological outcomes. In addition, the authors described that preoperative serum levels of Ca 19.9 were independently related to reported survival up to 4 years, instead of 5 years.

Unfortunately, it has been widely reported that even if nodal positivity is one of the main factors influencing the prognosis of PDAC patients, there are limited possibilities for identifying positive lymphnodes with common radiological investigations. These tools offer information regarding the size and the morphology of the lymphnodes, but, as reported by Shin, the accuracy of preoperative radiologic imaging to assess N+ is poor (36).

On this basis, one of the challenges to win in the fight against this dreadful disease is the identification of tools able to predict the nodal status before any treatment. The availability of tools with the ability of identifying lymph node metastases in R-PDACs would allow the selection of a subgroup of patients who, even if resectable, could benefit from an NAT instead of upfront surgery in order to achieve better of the prognosis.

In this present experience, we reported that Ca 19.9, the only approved marker in clinical practice for pancreatic cancer, is able to predict the N+ in R-PDACs.

According to ROC curve analysis results, the optimal cut-off of Ca 19.9 to predict N+ in our series was 33 U/ml. As this cut-off is in line with the cut-off of 37 U/ml, suggested in literature (37) and used in our laboratory for PDAC, our results strengthen the reliability of this last value.

These findings are in part in agreement with those reported by Mattiucci (22).

Mattiucci et al. reported that elevated preoperative Ca 19.9 is significantly associated with nodal status (p <0.001), without impacting R. Notably, in addition to what Mattiucci already highlighted, based on our findings, the predictive efficacy of Ca 19.9 regarding N+ would be lost in the presence of hypoalbuminemia.

Taking into account that a more advanced disease should be associated with worse nutritional status (28), and considering that other authors recently reported how elevated levels of Ca 19.9 are associated with worse prognosis in stage III PDAC and to lower levels of serum albumin (31), the link between SAL and Ca 19.9 was investigated in the present study.

Subsequently, this study revealed that, although there was a significant link between Ca 19.9 and lymph node involvement, and therefore with a more advanced stage of disease, this significance was not confirmed in patients with low albumin levels.

Therefore, unlike what Zhang reported, our results do not seem to confirm that a worse nutritional state corresponds to a more advanced disease; in fact, according to our findings, we could hypothesize that albumin may affect the predictive capacity of Ca 19.9.

Excluding patients with hypoalbuminemia, which our study revealed to correspond with the oldest patients and to those with higher ASA scores, and in disagreement with Mattiucci and Zhang (22, 31) and colleagues, Ca 19.9 at the cut-off of >418 U/ml could also be a predictor with high specificity (87%) and R+.

Taking into account that the margin status positivity is one of the predictors of early recurrence after pancreatic cancer surgery, the utility of Ca 19.9 has already been demonstrated in a previously published paper on this topic. Fiore et al. in their experience, reported that Ca 19.9 levels higher than 698 U/ml were able to identify early progression of pancreatic cancer in patients who had undergone radical resection. In these patients, the odds to develop a recurrence were six times higher (23).

These findings disagree with what was reported by Mosquera in a retrospective review on 181 pancreatic cancer patients who had undergone radical pancreaticoduodenectomy, did not find any significant association between the R+ and preoperative levels of Ca 19.9 (38).

Notwithstanding the possible association between Ca 19.9 and margin status detected at ROC curve analysis, these findings were not confirmed by the multivariate analysis.

For all these reasons, the clinical use of Ca 19.9 in daily practice cannot be taken into account to assess the risk of margin positivity after pancreatic surgery.

In view of the trend towards a significant correlation (p = 0.059) between higher rates of VR for Ca 19.9 levels >78 U/ml, it is our opinion that a more extensive series could confirm this link and therefore lead to a positive consideration of Ca 19.9 as also assisting in predicting more locally advanced disease in terms of local vascular invasion.

Nonetheless, it has to be taken into account that the decrease of Ca 19.9 levels has been used to plan arterial vascular resection for locally advanced pancreatic cancer after NAT, as well as to predict progression of the disease after treatment in advanced cases (39, 40).

To the best of our knowledge, this study would be the first reporting on the possible role of Ca 19.9 in predicting the need for VR in R-PDACs.

The effect of albumin on the predictive ability of Ca 19.9 reported in this experience is in line with the evidence that albumin improves the diagnostic accuracy of Ca 19.9 in detecting PDAC (26).

For instance, in the Herreros-Villanueva experience, biomarkers panel, including Ca 19.9 and albumin, showed higher sensitivity in detecting PDAC, particularly in its advanced stage.

Nonetheless, in 2014 Pant (27) reported how baseline albumin levels have a prognostic role in advanced PDAC treated with bevacizumab.

In our experience, serum albumin levels did not show any significant association with N, R+ and with the need for VR.

These results are in part in agreement with what was reported by Feng (41) who conducted a study on 201 patients affected by advanced pancreatic cancer.

The author noted that baseline albumin levels are not associated with patient prognosis.

Furthermore, albumin, an endogenous antioxidant, recognized as a risk factor for poor prognosis in pancreatic cancer (42), in our series was found to be normal in cases of tumor localized in the body or tail of the pancreas. These localizations, however, according to what was reported by Watanabe and Artinyan (43, 44), were found to be those burdened with worse prognosis.

Its retrospective design limits this study; moreover, it is not possible to exclude potential biases from a single-center experience and, patients considered eligible for the analysis were considered over a long time span.

The relation between Ca 19.9 and PDAC has been widely investigated in the literature. The majority of the studies currently published on this topic focus on the relation between Ca 19.9 and prognosis or progression after therapy or on the risk of recurrence of PDAC.

Only a minority of the studies focus on the relation between Ca 19.9 and NAT for resectable PDACs, with varying results and conclusions being reported.

Katz (45), in his study, concluded that the decision-making process regarding the use or not of neoadjuvant in potentially resectable PDAC must be essentially based on the clinical judgment of experts and on radiological staging, rather than on Ca 19.9 levels.

On the contrary, in 2016 researchers at the Mayo Clinic (46) demonstrated how early stage PDACs with high levels of Ca 19.9 need to be considered “biologically” borderline resectable, and therefore eligible for NAT. On this basis, guidelines suggesting neoadjuvant before surgery in resectable or borderline resectable pancreatic tumors with elevated Ca 19.9 levels have been drawn up.

More recently, these guidelines have been put into question. Authors, such as Kim in 2020 (47), studying the association between Ca 19.9 and oncological outcomes of resectable PDACs, were in agreement with Katz (45) that, despite the recommendations of the aforementioned guidelines, it is not possible to establish therapeutic strategy on the basis of Ca 19.9 values alone.

Our findings are the result of the analysis of a series of patients undergoing upfront surgery, based only on radiological evidence, and suggest the role of Ca 19.9 in improving PDAC staging, identifying a subgroup of patients that, although considered radiologically resectable, had a more advanced disease, and therefore would have benefited from NAT.

Our results, in contrast with those reported by Kim (47) and only partially in agreement with those of Bergquist (46) and Katz (45), could provide an explanation for the differences reported to date on the reliability of Ca 19.9 in defining radiologically resectable PDACs as “biologically borderline resectable”.

Notably, we found that serum albumin levels influence the ability of Ca 19.9 in predicting N. Based on this new outcome, it is our aim to assess a multi-center study in order to validate the role of Ca 19.9 in radiologically resectable PDAC patients with normal serum albumin levels.

The strengths of this study lie in the analysis of unselected PDAC who received upfront surgery, and in the fact that all the procedures were performed by the same expert pancreatic surgeon with well-standardized surgical technique avoiding possible bias due to different types of lymphadenectomy.

The results obtained could represent a step forward in using Ca 19.9 as a simple, cost-effective, and user-friendly tool in the therapeutic choice for patients affected by PDAC with normal albumin levels.

## Conclusions

In this study, increased preoperative levels of Ca 19.9 predicted the presence of nodal involvement in patients affected by PDAC and with normal albumin levels who received radical resection. In addition, according to our findings, the margin status and the need for VR could also be predicted by analyzing preoperatively the levels of Ca 19.9.

These represent the results of a pilot investigation that could lay the basis for a validation study carried out in a multi-center context in order to confirm these findings.

## Data Availability Statement

The raw data supporting the conclusions of this article will be made available by the authors, without undue reservation.

## Ethics Statement

The studies involving human participants were reviewed and approved by Ethical Committee of the University Campus Bio-Medico of Rome. Written informed consent for participation was not required for this study in accordance with the national legislation and the institutional requirements.

## Author Contributions

Conceptualization, AC, DC, and VV. Methodology, AC, DC, and MF. Formal analysis, AC and SA. Data curation, TF, AC, and SA. Writing—original draft preparation, AC, DC, TF, VV, SA, RC, and MF. Writing review and editing, AC, SR, SA, and RC. Supervision, SR, RC, and DC. All authors contributed to the article and approved the submitted version.

## Conflict of Interest

The authors declare that the research was conducted in the absence of any commercial or financial relationships that could be construed as a potential conflict of interest.
